# Type 2 diabetes mellitus increases long-term mortality risk after isolated surgical aortic valve replacement

**DOI:** 10.1186/s12933-019-0836-y

**Published:** 2019-03-15

**Authors:** Eilon Ram, Alexander Kogan, Shany Levin, Enrique Z. Fisman, Alexander Tenenbaum, Ehud Raanani, Leonid Sternik

**Affiliations:** 1 Department of Cardiac Surgery, Tel Aviv University, Tel Aviv, Israel; 2 Cardiac Surgery Intensive Care Unit, Tel Aviv University, Tel Aviv, Israel; 3 Sheba Medical Center, Tel Hashomer, Affiliated to the Sackler School of Medicine, Tel Aviv University, Tel Aviv, Israel

**Keywords:** Diabetes mellitus, Aortic valve replacement, Insulin

## Abstract

**Background:**

Diabetes mellitus (DM) adversely affects morbidity and mortality for major atherosclerosis-related cardiovascular diseases and is associated with increased risk for the development of aortic stenosis. Clinical data regarding the impact of DM on outcomes of patients undergoing aortic valve replacement (AVR) have revealed inconsistent results. The aim of the current study was to investigate and compare the impact of type 2 DM on short-, intermediate- and long-term mortality between DM and non-DM patients who undergo isolated AVR.

**Methods:**

We performed an observational study in a large tertiary medical center over a 14-year period (2004–2018), which included all patients who had undergone isolated AVR surgery for the first time. Of the 1053 study patients, 346 patients (33%) had type 2 DM (DM group) and were compared with non-DM (non-DM group) patients (67%). Short-term (in-hospital), intermediate (1- and 3-years), and late (5- and 10-years) mortality were evaluated. Mean follow-up of was 69 ± 43 months.

**Results:**

Short-term (in-hospital) mortality was similar between the DM compared with the non-DM group: 3.5% and 2.5% (p = 0.517). Intermediate-term mortality (1- and 3-year) was higher in the DM group compared with the non-DM group, but did not reach statistical significance: 8.1% vs. 5.7% (p = 0.169) and 12.1% vs. 8.3% (p = 0.064) respectively. Long-term (5- and 10-year) mortality was significantly higher in the DM group, compared to the non-DM group: 19.4% vs. 12.9% (p = 0.007) and 30.3% vs. 23.5% (p = 0.020) respectively. Among the 346 DM patients, 55 (16%) were treated with insulin and 291 (84%) with oral antiglycemic medication only. Overall in-hospital mortality among insulin-treated DM patients was 7.3% compared with 2.7% among non insulin-treated DM patients (p = 0.201). Long-term mortality was higher in the subgroup of insulin-treated DM patients compared with the subgroup of non-insulin treated DM patients with an overall mortality rate of 36.4% vs. 29.2% (p = 0.039). Furthermore, predictors for late mortality included DM (HR 1.39 CI 1.03–1.86, p = 0.031) and insulin treatment (HR 1.76 CI 1.05–2.94, p = 0.033), as demonstrated after adjustment for confounders by multivariable analysis.

**Conclusions:**

Type 2 DM is an independent predictor for long-term mortality after isolated AVR surgery.

## Introduction

Aortic valve stenosis (AS), the most commonly acquired valve disorder, is emerging as a new epidemic in the western world due to ageing populations [1].

Diabetes mellitus (DM) adversely affects morbidity and mortality for major atherosclerosis-related cardiovascular diseases [2]. Following cardiac surgery and particularly coronary artery bypass surgery, patients with DM have been shown to suffer higher rates of adverse events including higher mortality rates [3–5]. Furthermore, DM is associated with increased risk for the development of AS [6, 7], and was found to be the second most significant factor associated with AS after hypertension [8]. Aortic valves from diabetic patients with AS who require valve replacement have shown more calcification, with a higher grade of mineralization than non-diabetic patients [9].

Clinical data regarding the impact of DM on outcomes of patients undergoing aortic valve replacement (AVR) have revealed inconsistent results [10, 11]. The aim of the current study was to investigate and compare the impact of type 2 DM on short-, intermediate- and long-term mortality between DM and non-DM patients who undergo isolated AVR for the first time.

## Methods

### Study design and population

We performed a retrospective, observational study that included prospectively-collected data from a large tertiary university hospital. Between 01.09.2004 and 31.06.2018 a total of 1053 patients underwent their first isolated AVR. Of them 346 (33%) suffered from type 2 DM (DM group) and 707 (67%) had no DM (non-DM group). DM type 2 was defined according to the American Diabetes Association as: (a) hemoglobin A1C ≥ 6.5%; (b) fasting plasma glucose levels ≥ 126 mg/dL (7 mmol/L); (c) classic symptoms of hyperglycemia or a hyperglycemic crisis, a random plasma glucose level ≥ 200 mg/dL (11.1 mmol/L) [12]; or (d) currently on pharmacologic treatment (oral antihyperglycemic drugs and/or insulin). The results of the 346 diabetic patients (55 with and 291 without insulin therapy) were analyzed by the use of insulin treatment. The study was approved by the Sheba Medical Center Institutional Ethics Committee (Protocol No 4257). The requirement for informed consent was waived because of the retrospective nature of the study.

### Surgical procedures and postoperative care

Standard cardiopulmonary bypass was established by cannulation of the ascending aorta and the right atrium or the right femoral artery and vein. Myocardial protection was achieved by using antegrade and/or retrograde cold blood cardioplegia.

After surgery all patients were admitted to the intensive care unit (ICU) directly from the operating room. Following discharge from the ICU, patients were transferred either to a step-down unit or directly to the floor, from where they were discharged either to their home or to a rehabilitation facility. In the operating room and in the ICU, patients from both the DM and non-DM groups received intravenous continuous infusion of regular insulin according to the Society of Thoracic Surgeons practice guideline series [13]. After discharge from the ICU, the non-DM patients received no insulin nor any other hypoglycemic medication, while the DM patients continued to receive preoperative antiglycemic treatment (per oral drugs and insulin), as soon as they began to eat.

### Data collection and follow-up

All hospital data were ascertained by hospital chart review, telephone contact, and clinical follow-up. Data included: demographic parameters, medical history, chronic and peri-procedural medical treatment, echocardiography measurements, procedural information, and outcome measures. Mortality data were ascertained from the Israeli Ministry of Interior, Population Register through November 2018. Mean follow-up was 69 ± 43 months and was completed for 98% of the patients.

### *S*tatistical analysis

Data are presented as mean ± standard deviation. Continuous variables were tested with the Kolmogorov–Smirnov test for normal distribution. Categorical variables are given as frequencies and percentages. A Chi square test was used for comparison of categorical variables between the DM and non-DM groups and between oral antihyperglycemic-treated and insulin-treated DM patients. A Student’s t-test was performed for comparison of normally distributed continuous variables and Mann–Whitney U test for non-normal distribution.

Cox regression analysis was used to identify factors associated with long-term mortality adjusted to patient’s age. Candidate covariates are provided in Table 1. A Cox proportional hazard model was constructed to assess the association between DM and long-term mortality. Variables that were associated with long-term mortality adjusted to age were included in the regression model. In addition, we included pre-specified clinically significant variables in the model. The variables included in the final model were: age, gender, peripheral vascular disease, chronic obstructive pulmonary disease, New York Heart Association functional class III–IV and hyperlipidemia. In addition, Kaplan–Meier survival analysis was performed to compare long-term mortality among the subgroup of DM patients, with statistical differences tested by the log-rank test.

**Table 1:** Patient characteristics

	Diabetes (N = 346)		Non-diabetes (N = 707)	p-value Insulin vs. non-insulin	p-value DM vs. non-DM
	Insulin treatment (N = 55)	Non-insulin treatment (N = 291)			
Age (years)	69 ± 8	70 ± 10	67 ± 14	0.339	< 0.001
Gender (male)	30 (55%)	161 (55%)	361 (51%)	1.000	0.240
Hypertension	50 (91%)	257 (88%)	447 (63%)	0.745	< 0.001
Previous MI	8 (15%)	30 (10%)	51 (7%)	0.493	0.052
COPD	12 (22%)	33 (11%)	49 (7%)	0.057	0.002
Dialysis	3 (6%)	5 (2%)	8 (1%)	0.229	0.229
Hyperlipidemia	50 (91%)	240 (83%)	382 (54%)	0.174	< 0.001
Smoking	8 (15%)	79 (27%)	115 (16%)	0.071	0.001
PVD	6 (11%)	23 (8%)	28 (4%)	0.637	0.005
Prior CVA/TIA	3 (6%)	23 (8%)	50 (7%)	0.724	0.894
Neurological deficit	2 (4%)	7 (2%)	18 (2%)	0.949	1.000
Atrial fibrillation	5 (9%)	12 (4%)	38 (5%)	0.221	0.866
BMI (mean)	37 ± 47	32 ± 32	28 ± 14	0.345	0.004
Obesity	23 (42%)	109 (38%)	147 (21%)	0.646	< 0.001
Standard EuroSCORE I	7.9 ± 12.6	5.9 ± 2.6	5.6 ± 2.6	0.021	0.022
NYHA FC III–IV	30 (55%)	115 (40%)	240 (35%)	0.072	0.029
Ejection fraction (%)	55 ± 12	56 ± 11	57 ± 11	0.341	0.125
Pulmonary hypertension	7 (13%)	16 (6%)	31 (4%)	0.093	0.157
Bicuspid aortic valve	5 (9%)	32 (11%)	109 (15%)	0.856	0.047

*DM* Diabetes mellitus, *MI* myocardial dysfunction, *COPD* chronic obstruction pulmonary disease, *PVD* peripheral vascular disease, *CVA* cerebral vascular accident, *TIA* transient ischemic attack, *BMI* body mass index, *NYHA FC* New York Heart Association functional class

Statistical significance was assumed when the null hypothesis could be rejected at p < 0.05. All p-values are the results of two-sided tests. Statistical analyses were conducted using R (version 3.4.1) [14].

## Results

### Baseline characteristics

During the 14-year study period, 1053 patients underwent isolated AVR and were included in the study. There were 707 patients (67%) in the non-DM group and 346 patients (33%) in the DM group (Table 1). Of them, 291 patients (84%) were treated with oral antihyperglycemic medications and 55 patients (16%) were treated with insulin (Table 1). Compared with the non-DM group, the DM group had significantly more co-morbidities as shown in Table 1. Operative date was similar between the DM and non-DM groups (Table 2).

**Table 2:** Operative data

	Diabetes (N = 346)	Non-diabetes (N = 707)	p-value
Minimally invasive			0.218
Mid-sternotomy	286 (83%)	611 (86%)	
J-sternotomy	50 (14%)	76 (11%)	
Right mini-thoracotomy	10 (3%)	20 (3%)	
Valve prosthesis			0.084
Biological	317 (92%)	621 (88%)	
Mechanical	29 (8%)	86 (12%)	
Valve size			0.237
18	1 (0.3%)	1 (0.1%)	
19	24 (7%)	58 (8.3%)	
20	8 (2.3%)	20 (2.9%)	
21	120 (35.1%)	232 (33.2%)	
22	14 (4.1%)	26 (3.7%)	
23	97 (28.4%)	148 (21.2%)	
24	13 (3.8%)	29 (4.1%)	
25	37 (10.8%)	125 (17.9%)	
26	7 (2%)	14 (2%)	
27	16 (4.7%)	38 (5.4%)	
28	1 (0.3%)	2 (0.3%)	
29	4 (1.2%)	5 (0.7%)	
33	0 (0%)	1 (0.1%)	
Cross-clamp time (min)	63 ± 20	64 ± 45	0.761
Cardiopulmonary bypass time (min)	115 ± 44	114 ± 44	0.715
Total operative time (min)	229 ± 50	226 ± 48	0.361

### Surgical procedures

A median sternotomy approach was performed in 897 patients (85.2%); a partial sternotomy (J-sternotomy) was performed on 126 patients (12%), and a right mini-thoracotomy on 30 patients (2.8%). 938 patients (89%) received a biological prosthesis and 115 (11%) a mechanical prosthesis.

### Mortality

Short-term (in-hospital) mortality was similar between the DM compared with the non-DM patients: 3.5% vs. 2.5%, p = 0.517. While overall intermediate-term mortality (1- and 3-year) were somewhat higher in the DM compared with the non-DM patients, it did not reach statistical significance: 8.1% vs. 5.7%, p = 0.169; 12.1% vs. 8.3%, p = 0.064; respectively.

**Fig. 1:**
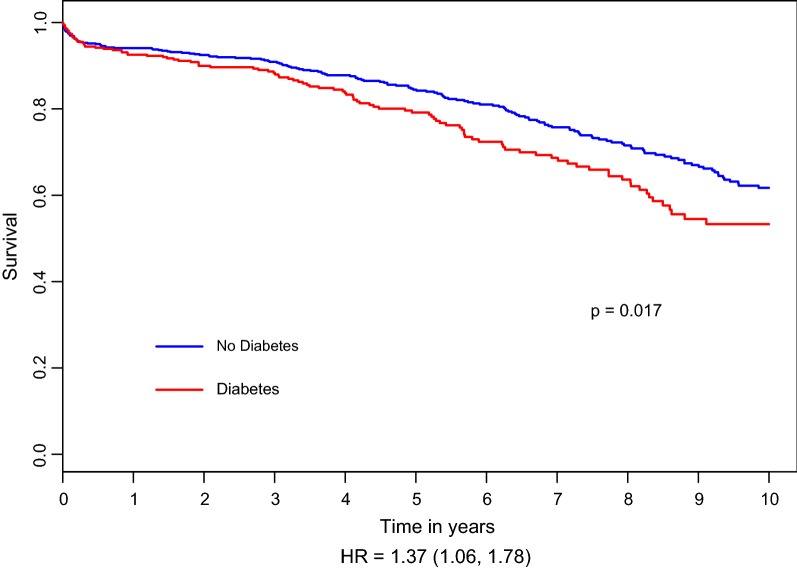
Hazard plot for survival at 10-year by the presence of diabetes mellitus, with propensity score adjustment. The covariates included in the model were: age, gender, PVD, COPD, NYHA functional class III–IV and hyperlipidemia. *HR* Hazard ratio, *PVD* peripheral vascular disease, *COPD* chronic obstruction pulmonary disease, *NYHA* New York Heart Association

Comparison between DM and non-DM patients regarding late mortality revealed a significant long-term advantage in favor of the non-DM patients (Fig. 1). The 5- and 10-year mortality rates were higher in patients with, compared to those without DM: 19.4% vs. 12.9%, p = 0.007 and 30.3% vs. 23.5%, p = 0.020; respectively (Table 3 and Fig. 1).

**Table 3:** Hazard ratios for mortality at 1-, 3-, 5-, and 10-years, adjusted for age

	1-year			3-year			5-year			10-year		
	HR	95% CI	p-value	HR	95% CI	p-value	HR	95% CI	p-value	HR	95% CI	p-value
Diabetes	1.33	0.82–2.15	0.250	1.37	0.92–2.04	0.117	1.47	1.07–2.01	0.018	1.44	1.12–1.84	0.004
Gender (male)	1.51	0.93–2.45	0.097	1.55	1.04–2.32	0.031	1.31	0.96–1.79	0.093	1.12	0.88–1.43	0.348
Obesity	0.72	0.39–1.32	0.288	0.92	0.58–1.45	0.713	0.87	0.59–1.26	0.454	0.95	0.72–1.26	0.737
Hypertension	0.67	0.4–1.12	0.129	0.67	0.44–1.03	0.068	0.83	0.57–1.19	0.307	0.91	0.68–1.21	0.521
PVD	2.45	1.21–4.95	0.013	2.90	1.67–5.02	0.000	2.58	1.61–4.14	0.000	2.08	1.4–3.07	0.000
Previous MI	1.61	0.82–3.17	0.168	1.53	0.87–2.7	0.143	1.32	0.82–2.11	0.253	1.19	0.82–1.71	0.356
Hyperlipidemia	0.49	0.3–0.79	0.004	0.63	0.42–0.93	0.022	0.69	0.5–0.95	0.024	0.79	0.62–1.01	0.059
Smoking	0.59	0.29–1.18	0.137	0.95	0.58–1.56	0.850	0.9	0.6–1.34	0.602	0.96	0.71–1.31	0.812
COPD	2.05	1.07–3.9	0.030	2.18	1.3–3.68	0.004	2.46	1.62–3.72	0.000	2.42	1.72–3.41	0.000
NYHA FC III–IV	1.23	0.76–2	0.393	1.24	0.83–1.84	0.289	1.32	0.96–1.81	0.086	1.28	1.01–1.64	0.045
Ejection fraction	0.96	0.95–0.98	0.000	0.97	0.95–0.98	0.000	0.97	0.96–0.99	0.000	0.98	0.97–0.99	0.000

*PVD* peripheral vascular disease, *MI* myocardial dysfunction, *COPD* chronic obstruction pulmonary disease, *NYHA FC* New York Heart Association functional class

Multivariable analysis demonstrated that predictors for late mortality include DM (p = 0.031), older age, with 7% increased odds for mortality per 1-year increment in age (p < 0.001), chronic obstructive pulmonary disease (p = 0.008), and lower ejection fraction (p = 0.003) (Table 4).

**Table 4:** Cox regression analysis

	Univariable analysis			Multivariable analysis		
	HR	CI	p-value	HR	CI	p-value
Age	1.08	1.06–1.09	< 0.001	1.07	1.06–1.09	<0.001
Diabetes	1.55	1.21–1.97	< 0.001	1.39	1.03–1.86	0.031
PVD	2.36	1.6–3.49	< 0.001	1.35	0.81–2.25	0.252
COPD	2.19	1.57–3.05	< 0.001	1.89	1.19–3.02	0.008
NYHA FC III-IV	1.53	1.2–1.93	< 0.001	1.03	0.76–1.39	0.846
Ejection fraction	0.98	0.96–0.99	< 0.001	0.98	0.97–0.99	0.003

Predictors for late mortality by univariable and multivariable analysis

*PVD* Peripheral vascular disease, *COPD* chronic obstruction pulmonary disease, *NYHA FC* New York Heart Association functional class, *HR* hazard ratio, *CI* confidence interval

### Subgroup analysis

Among the 346 DM patients, 55 (16%) were treated with insulin and 291 (84%) with oral antiglycemic medication only (Table 1). Overall in-hospital mortality among insulin-treated DM patients was 7.3% compared with 2.7% among non insulin-treated DM patients (p = 0.201). Intermediate-term (1- and 3-year) mortality was higher in the insulin-treated DM group compared with the non insulin-treated DM group, but did not reach statistical significance: 12.7% vs. 7.2% (p = 0.269) and 16.4% vs. 11.3% (p = 0.412) respectively.

**Fig. 2:**
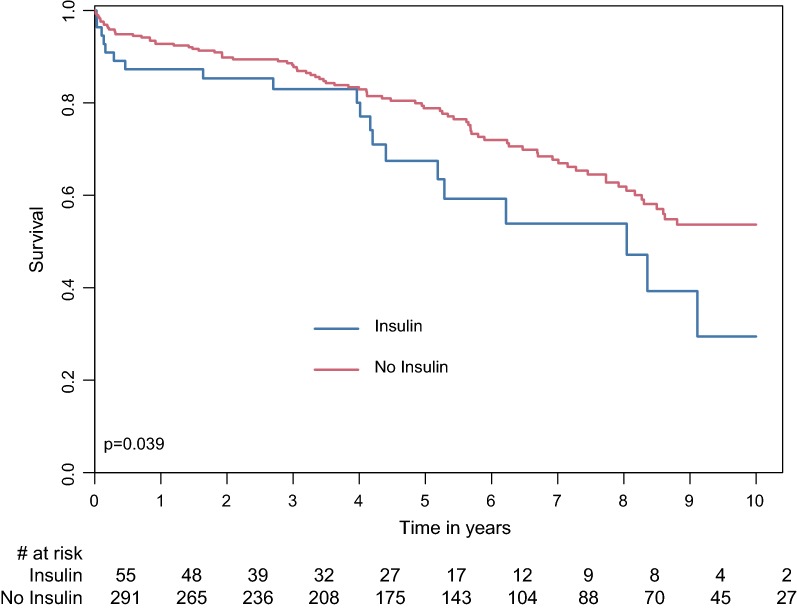
Survival rate in the DM group by insulin treatment. *DM* Diabetes mellitus

Furthermore, long-term mortality was higher in the subgroup of insulin-treated DM patients compared with the subgroup of non-insulin treated DM patients with an overall mortality rate of 36.4% vs. 29.2% (p = 0.039, Fig. 2). Among the DM patients, predictors for late mortality were insulin treatment (HR 1.76 95% CI 1.05–2.94, p = 0.033), older age, with 6% increased odds for mortality per 1-year increment in age (p < 0.001), and chronic obstructive pulmonary disease (HR 1.89 95% CI 1.12–3.19, p = 0.018).

## Discussion

This study, carried out in a contemporary cohort of patients who underwent their first isolated AVR, demonstrates several important implications regarding the impact of type 2 DM on in-hospital, mid- and long-term mortality. We have shown that long-term mortality was higher in DM patients than in non-DM patients, and that mortality rate was affected by the diabetic treatment strategy with worse outcomes in patients treated with insulin as compared with patients not treated with insulin.

While the impact of diabetes on short-term mortality after AVR remains controversial, DM has been included in the Society of Thoracic Surgeons (STS) risk score [15] as a marker of poor prognosis after cardiac surgery. EuroSCORE II is a very good predictor of in-hospital mortality after cardiac surgery and can be safely be used for quality assurance and risk assessment [16] and insulin-treated DM has been specifically included in it [17]. Both the STS score and the EuroSCORE II were validated to predict 30-day mortality after cardiac surgery [15, 17]. López-de-Andrés et al. [18] reported a significantly lower in-hospital mortality rate among DM patients (3.9–8.9%) than among non-DM patients (5.1–7.8%) (p < 0.001), and Abramowitz et al. [19] reported a lower 30-day mortality rate among DM compared with non-DM patients (5% vs. 5.9%, p < 0.001), while Linke et al. [20] found no differences in 30-day mortality between DM and non-DM patients: 6.2% vs. 7.5%. Anyway, data are controversial since DM has been found to be significantly and consistently associated to higher in-hospital mortality in a huge Spanish population after major cardiovascular events [21], and also Mendez-Bailon et al. [22] reported a lower in-hospital mortality rate in patients with, compared to those without DM (4.4% vs. 6.3%, p < 0.01). About a third of our study patients were diabetic, with 55 of them (16%) receiving insulin treatment.

We report here that in-hospital mortality among DM and non-DM patients, was 3.5% and 2.5%. In the non-insulin (N = 291) compared with the insulin-treated (N = 55) subgroup of patients, in-hospital mortality was 2.7% and 7.3%, p = 0.201. While our findings were not statistically significant, the difference reported by us could be clinically relevant. Whereas our small sample size was underpowered to reach conclusive results, a larger cohort might have shown significantly higher early mortalities among DM patients, particularly in those on insulin therapy.

However, the impact of diabetes on mid- and long-term mortality after isolated AVR surgery has been consistent in several reports. Linke et al. [20] found significantly higher 1- and 3-year mortality rates between DM and non-DM patients: 21.6% vs. 20.5% (p = 0.02) and 33.4% vs. 28.4% (p < 0.01). A post hoc analysis of the PARTNER trial, stratified according to the DM status of patients randomly assigned to undergo AVR, revealed a 1-year mortality rate of 27.4% in DM patients and 23.7% in non-DM patients [23]. At 1-year, Abramowitz et al. [19] reported that DM was significantly associated with a higher mortality hazard (HR 1.3 95% CI 1.13–1.49, p < 0.001). This association was stronger among insulin-treated patients (HR 1.57 95% CI 1.28–1.91, p < 0.001). We report here the results of a longer follow-up period than previously published, with a mean follow-up of 69 ± 43 months, demonstrating consistent results toward a worse rate of survival among DM patients, particularly those receiving insulin.

Diabetes mellitus is one of the major causes of heart failure in patients with reduced ejection fraction [24], and even in cases in which ejection fraction is preserved [25, 26]. In general, insulin-treated DM patients have more co-morbidities than non insulin-treated DM patients [5, 27, 28] and are prone to more revascularization procedures [16, 29]. The presence of insulin treatment as a marker for more rapid prosthetic valve deterioration remains debatable. Furthermore, its underlying biological mechanism has not yet been fully elucidated. Insulin may be related to the impact of a procoagulant imbalance, chronic exposure to high glucose levels, and direct effects of hyperinsulinemia. Further studies are needed to examine whether insulin-treated DM patients should be included in risk stratification algorithms for patients who undergo first-time AVR.

Aortic valve replacement and transcatheter aortic valve implantation (TAVI) are the only effective treatments for severe AS. Currently, however, TAVI is limited to moderate-high risk patients only, when the risk of TAVI is estimated to be lower than the risk of AVR, taking into consideration the fact that long-term results of TAVI are still unknown [30]. While we and others have reported that DM is a significant risk factor for late mortality after AVR [10], long-term mortality after TAVI for DM patients still needs further investigation.

Among the entire cohort of patients in our study who underwent isolated AVR, those with DM were older. One could surmise that, as in ischemic heart disease, DM patients present with less symptoms and therefore tend to be diagnosed later, resulting in treatment delays. However, we believe that this is not the case in DM AVR patients. Compared with DM patients, non-DM patients in our study had a significantly higher presence of bicuspid aortic valve, that tended to deteriorate at a higher rate, and therefore were operated on at an earlier stage of disease progression. We have shown that mortality was affected by the presence of DM regardless of patient age. While in the general population type 2 DM is associated with excess mortality compared with those without DM, with a HR of 1.15 at 5 years [31], we report here that DM had a greater impact on patients who underwent first-time isolated AVR (HR of 1.58 at 5 years).

### Limitations

There are a few limitations in our study. First, despite it being retrospective in design, data were collected prospectively and recorded in a well-defined database. Second, our study was conducted in a single-center cardiac surgery department. Third, we had no information regarding the main cause of death, the rate of cardiac events and data regarding prosthetic valve performance during the follow-up period. Analysis of cardiac events could reinforce the conclusion that DM provides less favorable results after AVR. The lack of information regarding the main cause of death weakens the conclusions of this study.

## Conclusions

Type-2 DM is an independent predictor of long-term (5- and 10-years) mortality after AVR. Mortality rates increased significantly when the diabetic treatment strategy included insulin. A larger study is required in order to examine weather DM has an impact on early mortality.

## Abbreviations

AS: aortic valve stenosis
AVR: aortic valve replacement
DM: diabetes mellitus
ICU: intensive care unit
TAVI: transcatheter aortic valve implantation
